# Serum Uric Acid Levels in Older Adults: Associations With Clinical Outcomes and Implications for Reference Intervals in Those Aged 70 Years and Over

**DOI:** 10.1002/acr.25621

**Published:** 2025-12-17

**Authors:** Amanda J. Rickard, Cammie Tran, Hans G. Schneider, Flavia M. Cicuttini, Anita E. Wluka, Ego Seeman, Johannes T. Neumann, Md Nazmul Karim, Zhen Zhou, Sultana Monira Hussain, David P. Q. Clark, Daniel Clayton‐Chubb, Andrew M. Tonkin, Lawrence J. Beilin, Robyn L. Woods, John J. McNeil

**Affiliations:** ^1^ Monash University Melbourne Australia; ^2^ Monash University and The Alfred Hospital Melbourne Australia; ^3^ University of Melbourne Melbourne Australia; ^4^ Monash University, Melbourne, Australia, and University Heart & Vascular Centre Hamburg Germany; ^5^ Monash University and University of Melbourne Melbourne Australia; ^6^ University of Western Australia Perth Australia

## Abstract

**Objective:**

Reports have linked both high and low serum uric acid (SUA) levels to adverse health outcomes. This study aimed to establish a reference interval for SUA in older adults and assessed its association with clinically relevant outcomes in relatively healthy, community‐dwelling individuals aged ≥70 years old.

**Methods:**

The study used data from the ASPirin in Reducing Events in the Elderly (ASPREE) trial. In Australia, 11,878 ASPREE participants had baseline SUA measurements (median age 74 years old). The study sample (n = 11,446; 55% women) comprised individuals with baseline SUA measurements, excluding those on urate‐lowering medication. The reference sample (n = 10,501; 55% women) was established after further exclusion of participants with impaired renal function, defined as an estimated glomerular filtration rate <45 mL/min/1.73m^2^. Reference intervals (2.5th and 97.5th percentile) were stratified by sex, and Cox proportional hazard models assessed associations between SUA levels and relevant clinical outcomes.

**Results:**

SUA reference intervals were 0.24 to 0.54 mmol/L for men and 0.19 to 0.48 mmol/L for women. After adjusting for potential confounders, no association was observed between SUA levels and all‐cause mortality, disability‐free survival, cardiovascular disease, major adverse cardiovascular events, cancer incidence and mortality, or dementia in either the study or reference samples. In women, however, low SUA levels were associated with an increased risk of fractures (hazard ratio 1.23; 95% confidence interval 1.04–1.46).

**Conclusion:**

Although previous reports have linked abnormal SUA levels to adverse health outcomes, our findings show no associations within the reference range, except for an increased fracture risk among women with low SUA levels.

## INTRODUCTION

Serum uric acid (SUA) is a clinical marker measured primarily for the diagnosis of gout and renal stones. An increasing number of epidemiologic studies, however, have reported associations between both high and low SUA levels and an increased risk of chronic diseases.^1^ Elevated SUA levels have been linked to the onset and progression of hypertension, cardiovascular disease (CVD), chronic kidney disease (CKD), cognitive decline, and higher all‐cause mortality.^2^ Conversely, low SUA levels have been associated with Alzheimer and Parkinson disease and increased mortality rate from all causes and CVD.^2^, ^3^ Given the growing clinical significance of SUA levels, identifying an optimal SUA range is increasingly relevant to clinical practice.

**SIGNIFICANCE & INNOVATIONS**

- This study established serum uric acid (SUA) reference intervals specifically for healthy adults aged ≥70 years old stratified by sex. The reference range was 0.24 to 0.54 mmol/L for men and 0.19 to 0.48 mmol/L for women.
- Despite prior evidence suggesting links between SUA levels and health risks, this study found no association between SUA levels and all‐cause mortality, disability‐free survival, cardiovascular events, cancer incidence and death, or dementia.
- A novel finding was that women with low SUA levels had a significantly higher risk of fractures, highlighting a sex‐specific health risk that may require further investigation.

In reference intervals proposed over the past two decades, there has been notable variation in both the upper and lower cutoff values (summarized in Supplementary Table 1). For men, the upper limits have ranged from 0.41 to 0.55 mmol/L, and the lower limits have ranged from 0.16 to 0.24 mmol/L.^4^, ^5^ In women, the upper limits have ranged from 0.35 to 0.44 mmol/L, and the lower limits have ranged from 0.13 to 0.17 mmol/L.^4^, ^5^, ^6^ This variability is likely influenced by factors such as difference in the age groups studied, the ethnicity of the populations examined, the prevalence of comorbidities, varying exclusion criteria, and differences in analytical methods across platforms.^7^

The purpose of this study was to establish a reference interval for SUA in a healthy, community‐dwelling population aged ≥70 years old, defined as individuals free of dementia, disability, and cardiovascular events. Additionally, we examined the association between SUA levels and the risk of death and a range of morbidity outcomes relevant to older adults. By focusing on an exclusively older, community‐dwelling population in which SUA measurements are common and chronic disease incidence is high, this study addresses an important gap in the literature. Data were sourced from the ASPirin in Reducing Events in the Elderly (ASPREE) study, which included an exclusively elderly cohort with extensive phenotyping and comprehensive long‐term follow‐up extending up to 11 years.

## METHODS

### Data source

**Figure 1:**
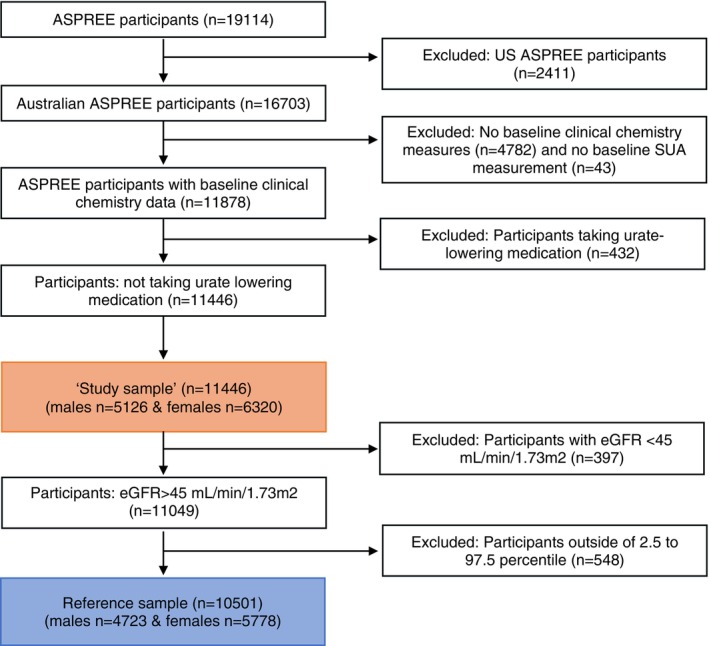
Consort flow‐chart

The ASPREE study was a randomized clinical trial of 19,114 participants from Australia and the United States. The protocol and primary results of the study have been published elsewhere.^8^ In brief, community‐dwelling individuals aged ≥70 years old were recruited between March 2010 and December 2014. Exclusion criteria included a history of CVD events, current CVD events, dementia, physical disability, or a chronic illness likely to cause death within the next five years. Details of the recruitment process, along with a full list of the inclusion and exclusion criteria, are provided in the Supplementary Methods. In Australia, 11,878 ASPREE participants provided nonfasting blood samples for the analysis of a range of biomarkers including SUA (Figure 1).

### Biospecimen collection and analysis

Blood samples were processed within four hours of collection, and serum was stored at −80°C for future analyses. SUA level was analyzed using a uricase/peroxidase colorimetric method (Abbott Alinity ci) with a coefficient of variation of 0.38% at 0.23 mmol/L and 1.2% at 0.57 mmol/L. Samples were analyzed at the Alfred Pathology Service, which is accredited by the National Association of Testing Authorities, adhering to the standards outlined in International Organization for Standardization 15189.^9^

### Study sample and reference sample

The initial study population (study sample) comprised all Australian ASPREE participants with a SUA measurement at baseline, excluding individuals taking urate‐lowering medication (Figure 1). The exclusion of individuals on allopurinol removed a group with pharmacologically lowered SUA levels, ensuring the remainder were reflective of physiologic SUA levels. The reference sample was established after further exclusion of participants with impaired renal function, defined as an estimated glomerular filtration rate (eGFR) <45 mL/min/1.73m^2^ (Supplementary Table 2 and Supplementary Figure 1). The reference interval was defined as the values falling between the 2.5th and 97.5th percentiles of the SUA distribution within the reference sample.

### Longitudinal outcomes

Longitudinal outcomes were determined via annual in‐person visits, medical record reviews, and six monthly phone calls. These included all‐cause mortality, disability‐free survival (DFS), CVD, major adverse cardiac events (MACE), cancer incidence and death, dementia, and fractures. DFS, a primary outcome of the ASPREE trial, is a composite endpoint defined by the first occurrence of death, dementia, or persistent physical disability.^8^ Death, dementia, and cancer were prespecified outcomes confirmed by adjudication committees comprised of specialist clinicians.^8^, ^10^, ^11^ CVD encompassed fatal coronary heart disease, nonfatal myocardial infarction, fatal or nonfatal stroke, or hospitalization for heart failure. MACE comprised a composite of fatal and nonfatal ischemic stroke, nonfatal myocardial infarction, or fatal coronary heart disease. A diagnosis of dementia was based on *Diagnostic and Statistical Manual of Mental Disorders, Fourth Edition* criteria,^12^ which included the presence of memory impairment, specific domains of cognitive disturbance, and an impact on functioning. Fractures included vertebral, hip, and other fractures that were confirmed by medical imaging.

### Analytical approach

To explore the cross‐sectional associations between SUA values and health measures, SUA levels in both the study sample and the reference sample were divided into quintiles. Quintile one represented the lowest 20% of SUA levels, quintile five represented the highest 20%, and quintile two to four were combined to represent the central 60%. Baseline characteristics, including self‐reported health issues, were assessed across these quintiles. Continuous variables were presented as median (interquartile range [IQR]) or mean (SD) and compared using the Kruskal–Wallis or analysis of variance test as appropriate. Categorical variables were expressed as proportions and compared using a chi‐square test.

Key health measures assessed included smoking status (current and former), alcohol use, body mass index (BMI), physical activity (self‐reported Lifestyle Interventions and Independence for Elders (LIFE) Disability questionnaire with low physical activity defined as “No walking outside the home or walked outside home but longest amount of time walked without sitting down to rest was less than 10 minutes”), and frailty status defined from five measures adapted from the Fried frailty domains (body weight, strength, exhaustion, walking speed, and physical activity).^13^ Frailty status was stratified according to the number of domains affected, that is, prefrail (one or two domains) and frail (three, four, or five domains). Hypertension was defined as a systolic blood pressure ≥140 mm Hg, a diastolic blood pressure ≥90 mm Hg, or a current use of antihypertensive medication. Other covariates included hyperlipidaemia, diabetes, albuminuria (urinary albumin/creatinine ratios), eGFR (CKD‐epidemiology estimating equation derived from serum creatinine) and abnormal liver enzymes (gamma‐glutamyl transpeptidase [GGT]).

To examine the association between SUA levels and clinical outcomes, Cox proportional hazards models were used to estimate cause‐specific hazard ratios (HR) and 95% confidence intervals (CI). Quintiles two to four were the reference. ASPREE clinical outcomes included all‐cause mortality, DFS, CVD, MACE, stroke, cancer incidence and death, dementia, and fractures. Analyses were conducted using both unadjusted and fully adjusted models. The fully adjusted model accounted for age (continuous), BMI (continuous), smoking status (categorical: current or former), current alcohol use (categorical), physical activity level (categorical), hypertension (categorical), diabetes (categorical), high‐density lipoprotein cholesterol (HDL‐C; continuous), non–HDL‐C (continuous), triglycerides (continuous), eGFR (continuous), and GGT (categorical: greater than 50 U/L). The statistical analyses were performed using Stata software version 17.0 (StataCorp LLC).

Ethics approval for the principal ASPREE trial was obtained from the Monash University Human Research Ethics committee (2006/745MC and CF11/1100) and for the ASPREE Healthy Ageing Biobank from the Alfred Hospital Ethics Committee (18/08). Written consent was obtained from all participants.

## RESULTS

### Study population

The SUA reference intervals for this older White population were established as 0.24 to 0.54 mmol/L for men and 0.19 to 0.48 mmol/L for women. A comparison of SUA levels from men and women using the Tukey‐Kramer test showed that men had significantly higher mean SUA levels (0.38 mmol/L; SD 0.08) than women (0.32 mmol/L; SD 0.08; Supplementary Table 3 and Supplementary Figure 2). Given this sex difference, all analyses were conducted separately for men and women. Of the 11,878 Australian participants who provided a blood sample at baseline, 432 individuals on urate‐lowering medication were excluded. The resulting study sample included 11,446 participants. The reference sample was further refined by excluding 397 participants with an eGFR below 45 mL/min/1.73m^2^, yielding a final sample of 10,501 participants.

The study sample (n = 11,446) had a median age of 74 years and consisted of 6,320 (55%) women (Table 1). Median SUA concentrations were 0.38 mmol/L (IQR 0.33–0.43) for men and 0.31 mmol/L (IQR 0.26–0.36) for women. The reference sample (n = 10,501) had a median age of 74 years and consisted of 5,778 (55%) women (Table 1). Median SUA concentrations were 0.37 mmol/L (IQR 0.33–0.42) for men and 0.31 mmol/L (IQR 0.26–0.35) for women. Among men, 2,605 participants (55.2%) were identified as current or former smokers, and 4,020 (85%) reported current alcohol consumption. In contrast, among women, 2,009 participants (35%) were current or former smokers, and 4,369 (76%) reported current alcohol consumption. The mean BMI was 28 for both men and women, and the waist‐to‐height ratio was 0.59 for men and 0.58 for women.

**Table 1:** Baseline participant characteristics according to SUA quintiles in both study and reference sample, stratified by sex*

	Study sample				Reference sample			
	Total	SUA quintiles			Total	SUA quintiles		
		Q1	Q2–4	Q5		Q1	Q2–4	Q5
Men^a^								
SUA, median (IQR), mmol/L	0.38 (0.33–0.43)	0.28 (0.26–0.30)	0.38 (0.35–0.41)	0.49 (0.46–0.52)	0.37 (0.33–0.42)	0.29 (0.27–0.31)	0.38 (0.35–0.40)	0.47 (0.45–0.50)
Age, median (IQR), y	73.8 (71.6–77.2)	73.8 (71.7–77.6)	73.8 (71.6–77.2)	73.8 (71.8–76.9)	73.7 (71.6–77.1)	73.9 (71.7–77.0)	73.7 (71.6–76.5)	73.6 (71.6–76.5)
Current or former smoker, n (%)	2,842 (55.4)	536 (51.6)	1,704 (55.3)	602 (59.9)	2,605 (55.2)	590 (52.6)	1,453 (54.5)	562 (60.1)
Current alcohol use, n (%)	4,356 (85.0)	868 (83.5)	2,607 (84.6)	881 (87.7)	4,020 (85.1)	929 (82.8)	2,264 (84.9)	827 (88.4)
Vegetarian diet, n (%)	63 (1.34)	22 (2.33)	36 (1.27)	5 (0.54)	59 (1.36)	20 (1.96)	33 (1.35)	6 (0.70)
BMI, mean (SD)	27.8 (3.73)	26.7 (3.44)	27.8 (3.59)	29.2 (3.97)	27.8 (3.7)	26.8 (3.5)	27.7 (3.5)	29.1 (3.9)
BMI, n (%)								
<21	80 (1.57)	32 (3.09)	40 (1.30)	7 (0.80)	69 (1.47)	24 (2.63)	38 (1.26)	7 (0.89)
21–29	3,792 (74.21)	850 (81.97)	2,319 (75.54)	623 (62.11)	3,523 (74.83)	742 (82.55)	2,274 (75.62)	497 (62.91)
≥30	1,238 (24.23)	155 (14.95)	711 (23.16)	372 (37.09)	1,116 (23.70)	135 (14.82)	695 (23.11)	286 (36.20)
Waist to height ratio, mean (SD)	0.59 (0.06)	0.57 (0.06)	0.59 (0.06)	0.61 (0.06)	0.59 (0.06)	0.57 (0.06)	0.59 (0.06)	0.61 (0.06)
Moderate to high physical activity, n (%)	3,467 (67.6)	714 (68.7)	2,141 (69.5)	612 (60.9)	3,216 (68.1)	774 (69.0)	1,854 (69.5)	588 (63.0)
Frailty status, n (%)								
Not frail	3,320 (64.8)	683 (65.7)	2,012 (65.3)	625 (62.2)	3,093 (65.5)	747 (66.6)	1,748 (65.6)	598 (64.0)
Prefrail or frail	1,806 (35.2)	356 (34.3)	1,070 (34.7)	380 (37.8)	1,630 (34.5)	375 (33.4)	918 (34.4)	337 (36.0)
Hypertension, n (%)	3,849 (75.1)	718 (69.1)	2,283 (74.01)	848 (84.4)	3,511 (74.3)	766 (68.3)	1,984 (74.4)	761 (81.4)
Antihypertensive medication, n (%)	2,437 (51.0)	388 (40.5)	1,393 (48.8)	656 (67.6)	2,179 (49.6)	416 (40.2)	1,195 (48.5)	568 (63.3)
Diuretics, n (%)	745 (14.5)	66 (6.35)	402 (13.0)	277 (27.6)	637 (13.5)	73 (6.51)	352 (13.2)	212 (22.7)
Non–HDL‐C, mean (SD), mmol/L	3.64 (0.89)	3.53 (0.93)	3.65 (0.88)	3.72 (0.87)	3.64 (0.89)	3.55 (0.91)	3.65 (0.87)	3.72 (0.88)
HDL‐C, mean (SD), mmol/L	1.41 (0.39)	1.51 (0.40)	1.40 (0.38)	1.33 (0.37)	1.41 (0.39)	1.50 (0.40)	1.39 (0.37)	1.35 (0.38)
Triglyceride, mean (SD), mmol/L	1.31 (0.67)	1.12 (0.55)	1.30 (0.63)	1.54 (0.81)	1.30 (0.66)	1.14 (0.56)	1.30 (0.63)	1.51 (0.78)
Diabetes, n (%)	584 (11.4)	125 (12.0)	340 (11.0)	119 (11.8)	512 (10.8)	122 (10.9)	284 (10.7)	106 (11.3)
Glucose, mean (SD), mmol/L	6.05 (1.71)	6.04 (2.00)	6.00 (1.60)	6.22 (1.69)	6.03 (1.66)	5.99 (1.84)	5.97 (1.54)	6.23 (1.74)
Chronic kidney disease, n (%)	778 (15.6)	72 (7.15)	408 (13.7)	298 (30.4)	592 (12.9)	74 (6.8)	326 (12.6)	192 (21.1)
eGFR, mean (SD), mL/min/1.73m^2^	73.3 (12.8)	78.6 (10.8)	73.5 (12.2)	66.9 (13.8)	74.2 (11.8)	78.0 (10.8)	74.0 (11.6)	70.1 (11.9)
uACR, median (IQR)	0.7 (0.4–1.7)	0.7 (0.4–1.4)	0.7 (0.4–1.4)	0.7 (0.4–1.7)	0.7 (0.4–1.3)	0.6 (0.4–1.3)	0.7 (0.4–1.3)	0.7 (0.4–1.6)
GGT, median (IQR), U/L	24 (18–34)	22 (16–30.5)	23 (17–33)	29 (21–43)	24 (18–34)	22 (17–31)	23 (17–33)	28 (21–41)
GGT, n (%)								
<50	4,542 (88.8)	953 (92.0)	2,770 (90.0)	819 (81.8)	4,197 (89.1)	1,024 (91.4)	2,404 (90.3)	769 (82.7)
>50	572 (11.2)	83 (8.0)	307 (10.0)	182 (18.2)	516 (11.0)	96 (8.6)	259 (9.7)	161 (17.3)
ALT, mean (SD), U/L	21.9 (11.5)	20.5 (13.6)	21.8 (10.2)	24.1 (12.6)	21.9 (11.3)	20.7 (13.4)	21.7 (10.4)	23.9 (10.9)
AST, mean (SD), U/L	22.4 (8.08)	22.1 (9.15)	22.2 (6.94)	23.5 (9.88)	22.4 (7.6)	22.0 (8.9)	22.2 (7.1)	23.3 (7.1)
Women^b^								
SUA, median (IQR), mmol/L	0.31 (0.26–0.36)	0.23 (0.21–0.24)	0.31 (0.28–0.34)	0.43 (0.40–0.46)	0.31 (0.26–0.35)	0.23 (0.21–0.24)	0.31 (0.28–0.34)	0.41 (0.39–0.44)
Age, median (IQR), y	73.9 (71.7–77.5)	73.6 (71.5–77.0)	73.8 (71.7–77.4)	74.6 (71.9–78.4)	73.8 (71.6–77.2)	73.6 (71.5–77.0)	73.7 (71.6–77.3)	74.3 (71.8–77.6)
Current or former smoker, n (%)	2,185 (34.6)	479 (35.0)	1,300 (34.3)	406 (35.1)	2,009 (34.8)	434 (35.7)	1,218 (34.4)	357 (35.1)
Current alcohol use, n (%)	4,731 (74.9)	1,010 (73.7)	3,892 (76.3)	829 (71.7)	4,369 (75.6)	904 (74.3)	2,709 (76.4)	756 (74.4)
Vegetarian diet, n (%)	166 (2.88)	49 (3.88)	99 (2.87)	18 (1.72)	150 (2.85)	41 (3.65)	95 (2.95)	14 (1.52)
BMI, mean (SD)	28.0 (5.04)	25.7 (4.18)	28.0 (4.76)	30.8 (5.46)	27.9 (4.9)	25.8 (4.2)	27.9 (4.8)	30.5 (5.3)
BMI, n (%)								
<21	313 (4.98)	140 (10.27)	154 (4.07)	19 (1.66)	275 (4.78)	114 (9.41)	151 (4.08)	10 (1.19)
21–29	4,045 (64.31)	1,035 (75.94)	2,494 (65.98)	516 (44.99)	3,762 (65.38)	923 (76.22)	2,443 (65.96)	396 (47.20)
≥30	1,932 (30.72)	188 (13.79)	1,132 (29.95)	612 (53.36)	1,717 (29.84)	174 (14.37)	1,110 (29.97)	433 (51.61)
Waist to height ratio, mean (SD)	0.58 (0.08)	0.55 (0.07)	0.58 (0.08)	0.63 (0.08)	0.58 (0.09)	0.55 (0.07)	0.58 (0.08)	0.62 (0.08)
Moderate to high physical activity, n (%)	3,827 (60.6)	920 (67.2)	2,305 (60.8)	602 (52.0)	3,525 (61.0)	808 (66.5)	2,151 (60.7)	599 (55.7)
Frailty status, n (%)								
Not frail	4,000 (63.3)	896 (65.4)	2,455 (64.7)	649 (56.1)	3,712 (64.2)	797 (67.1)	2,312 (65.2)	603 (59.4)
Prefrail or frail	2,320 (36.7)	474 (34.6)	1,338 (35.3)	508 (43.9)	2,066 (35.8)	419 (34.5)	1,234 (34.8)	413 (40.6)
Hypertension, n (%)	4,631 (73.3)	861 (62.9)	2,777 (73.2)	993 (85.8)	4,185 (72.4)	770 (63.3)	2,569 (72.5)	846 (83.3)
Antihypertensive medication, n (%)	3,416 (55.9)	571 (43.7)	1,983 (54.1)	862 (75.9)	3,028 (54.3)	509 (43.8)	1,810 (52.9)	709 (71.3)
Diuretics, n (%)	1,344 (21.3)	157 (11.5)	696 (18.7)	666 (57.6)	1,140 (19.7)	140 (11.5)	626 (17.7)	374 (36.8)
Non–HDL‐C, mean (SD), mmol/L	3.73 (0.96)	3.62 (0.92)	3.74 (0.96)	3.83 (1.00)	3.73 (0.95)	3.64 (0.92)	3.74 (0.96)	3.80 (0.97)
HDL‐C, mean (SD), mmol/L	1.74 (0.46)	1.86 (0.46)	1.74 (0.45)	1.57 (0.42)	1.74 (0.46)	1.86 (0.46)	1.75 (0.45)	1.59 (0.42)
Triglyceride, mean (SD), mmol/L	1.31 (0.61)	1.09 (0.49)	1.29 (0.57)	1.65 (0.70)	1.29 (0.58)	1.09 (0.49)	1.28 (0.56)	1.58 (0.64)
Diabetes, n (%)	491 (7.77)	71 (5.18)	269 (7.09)	151 (13.1)	445 (7.7)	63 (5.2)	248 (7.0)	134 (13.2)
Glucose, mean (SD), mmol/L	5.75 (1.43)	5.65 (1.62)	5.70 (1.32)	6.00 (1.54)	5.74 (1.43)	5.65 (1.61)	5.70 (1.33)	5.96 (1.53)
Chronic kidney disease, n (%)	1,125 (18.3)	88 (6.59)	574 (15.6)	463 (40.8)	828 (14.8)	77 (6.5)	475 (13.8)	276 (27.9)
eGFR, mean (SD), mL/min/1.73m^2^	72.9 (13.6)	78.8 (10.8)	73.4 (12.7)	64.4 (15.2)	74.1 (12.4)	78.6 (10.7)	74.0 (12.1)	68.9 (13.1)
uACR, median (IQR)	1.0 (0.6–1.7)	1.0 (0.6–1.9)	0.9 (0.6–1.7)	1.0 (0.6–1.9)	0.9 (0.6–1.7)	1.0 (0.6–1.8)	0.9 (0.6–1.6)	1.0 (0.5–1.8)
GGT, median (IQR), U/L	19 (17–34)	17 (13–24)	19 (14–28)	23 (17–34)	19 (14–28)	17 (13–24)	19 (14–28)	23 (17–34)
GGT, n (%)								
<50	5,816 (92.0)	1,304 (95.2)	3,485 (91.9)	1,027 (88.8)	5,319 (92.1)	1,152 (94.7)	3,255 (91.8)	912 (89.8)
>50	504 (8.0)	66 (4.8)	308 (8.1)	130 (11.2)	459 (7.9)	64 (5.3)	291 (8.2)	104 (10.2)
ALT, mean (SD), U/L	18.8 (9.84)	17.4 (6.95)	18.8 (10.1)	20.4 (11.6)	18.8 (9.5)	17.4 (7.1)	18.8 (10.1)	20.2 (9.5)
AST, mean (SD), U/L	21.3 (7.44)	21.1 (5.76)	21.3 (7.23)	21.7 (9.6)	21.3 (7.0)	21.1 (5.9)	21.3 (7.2)	21.4 (7.4)

* ALT, alanine aminotransaminase; AST, aspartate aminotransferase; BMI, body mass index; eGFR, estimated glomerular filtration rate; GGT, gamma‐glutamyl transferase; HDL‐C, high‐density lipoprotein cholesterol; IQR, interquartile range; Q, quintile; SUA, serum uric acid; uACR, urine albumin to creatinine ratio.

^a^ Study sample: total n = 5,126, Q1 n = 1,039, Q2–4 n = 3,082, Q5 = 1,005; reference sample: total n = 4,723, Q1 n = 1,122, Q2–4 n = 2,666, Q5 n = 935.

^b^ Study sample: total n = 6,320, Q1 n = 1,370, Q2–4 n = 3,793, Q5 n = 1,157; reference sample: total n = 5,778, Q1 n = 1,216, Q2–4 n = 3,546, Q5 n = 1,016.

### Baseline cross‐sectional analysis

Kernel density curves for SUA concentrations in the study and reference samples were analyzed by age, diuretics use, BMI, and alcohol consumption separately in men (Supplementary Figure 3) and women (Supplementary Figure 4). Although significant shifts in SUA distributions were observed when stratified by diuretic use, BMI, and alcohol intake, these differences were deemed insufficient to justify establishing separate reference intervals.

The baseline characteristics of the participants in the study and reference samples, stratified by SUA quintiles, are shown in Table 1. In both samples, male and female participants with higher SUA levels (quintile 5 [Q5]) were characterized by a higher BMI, greater waist‐to‐height ratio, increased total cholesterol–to–HDL‐C ratio, higher prevalence of hypertension, and greater frequency of impaired renal function (eGFR <60 mL/min/1.73m^2^) and elevated liver enzymes (alanine aminotransaminase [ALT], aspartate aminotransferase [AST], and GGT). Among women, higher SUA levels were also associated with a greater prevalence of diabetes.

### SUA levels and longitudinal outcomes

In the study and reference sample, over a median follow‐up ranging from 8.3 (IQR 6.9–9.4) to 8.6 (IQR 7.5–10.1) years, no significant relationships were identified between SUA quintiles and all‐cause mortality, CVD, MACE, or cancer mortality in the fully adjusted model (Table 2). Additionally, during a median follow‐up ranging from 6.0 (IQR 4.9–6.9) to 6.4 (IQR 5.3–7.5) years, no associations were observed between SUA quintiles and DFS, or dementia in either men or women. In women, low SUA levels (Q1) were associated with a higher risk of fractures in the fully adjusted model after a median follow‐up of 4.0 years (IQR 2.9–5.0; study sample: HR 1.23, 95% CI 1.04–1.46; reference sample: HR 1.22, 95% CI 1.02–1.46). Restricted spline curves demonstrated a nonlinear trend between SUA levels and risk of fractures in men and women (Supplementary Figure 5).

**Table 2:** Unadjusted and adjusted hazard ratios for association between SUA quintiles and clinical outcomes in both study and reference sample, stratified by sex*

	Study sample				Reference sample			
	Total	SUA quintiles			Total	SUA quintiles		
		Q1	Q2–Q4	Q5		Q1	Q2–Q4	Q5
Men^a^								
SUA, median (IQR), mmol/L	0.38 (0.33–0.43)	0.28 (0.26–0.30)	0.38 (0.35–0.41)	0.49 (0.46–0.52)	0.37 (0.33–0.42)	0.29 (0.27–0.31)	0.38 (0.35–0.40)	0.47 (0.45–0.50)
All‐cause mortality								
Number (rate per 1,000 PY)	881 (20.3)	171 (19.3)	519 (19.9)	191 (22.7)	788 (19.7)	189 (19.8)	434 (19.2)	165 (21.0)
Age adjusted HR (95% Cl)		0.89 (0.75–1.06)	1.00 (ref)	1.18 (1.00–1.39)		0.97 (0.82–1.16)	1.00 (ref)	1.15 (0.96–1.37)
Fully adjusted HR (95% Cl)^b^		0.85 (0.71–1.03)	1.00 (ref)	1.16 (0.97–1.39)		0.93 (0.78–1.12)	1.00 (ref)	1.13 (0.94–1.37)
Disability‐free survival^c^								
Number (rate per 1,000 PY)	812 (25.1)	156 (23.7)	484 (24.8)	172 (27.2)	740 (24.8)	169 (23.8)	417 (24.7)	154 (26.1)
Age adjusted HR (95% Cl)		0.90 (0.75–1.08)	1.00 (ref)	1.12 (0.94–1.34)		0.92 (0.77–1.10)	1.00 (ref)	1.11 (0.92–1.33)
Fully adjusted HR (95% Cl)^b^		0.88 (0.72–1.07)	1.00 (ref)	1.10 (0.91–1.33)		0.90 (0.74–1.08)	1.00 (ref)	1.07 (0.88–1.30)
Cardiovascular disease								
Number (rate per 1,000 PY)	624 (15.5)	120 (14.6)	363 (14.9)	141 (18.2)	568 (15.3)	130 (14.7)	310 (14.7)	128 (17.6)
Age adjusted HR (95% Cl)		0.94 (0.77–1.16)	1.00 (ref)	1.25 (1.03–1.52)		0.98 (0.80–1.20)	1.00 (ref)	1.24 (1.01–1.52)
Fully adjusted HR (95% Cl)^b^		1.07 (0.86–1.28)	1.00 (ref)	1.03 (0.83–1.28)		1.11 (0.90–1.38)	1.00 (ref)	1.09 (0.88–1.35)
MACE								
Number (rate per 1,000 PY)	505 (12.5)	94 (11.4)	295 (12.1)	116 (14.8)	463 (12.4)	105 (11.8)	253 (11.8)	105 (14.4)
Age adjustedHR (95% Cl)		0.91 (0.72–1.15)	1.00 (ref)	1.26 (1.01–1.56)		0.97 (0.77–1.21)	1.00 (ref)	1.24 (0.99–1.56)
Fully adjusted HR (95% Cl)^b^,		1.04 (0.82–1.33)	1.00 (ref)	1.04 (0.82–1.32)		1.10 (0.87–1.40)	1.00 (ref)	1.10 (0.87–1.41)
Dementia^c^								
Number (rate per 1,000 PY)	327 (7.97)	75 (8.98)	200 (8.10)	52 (6.50)	307 (8.11)	80 (8.86)	177 (8.29)	50 (5.07)
Age adjusted HR (95% Cl)		1.05 (0.81–1.37)	1.00 (ref)	0.82 (0.61–1.11)		1.04 (0.80–1.35)	1.00 (ref)	0.85 (0.62–1.16)
Fully adjusted HR (95% Cl)^b^		0.95 (0.71–1.27)	1.00 (ref)	0.93 (0.67–1.29)		0.98 (0.74–1.30)	1.00 (ref)	0.89 (0.64–1.25)
Cancer incidence								
Number (rate per 1,000 PY)	1,214 (32.1)	209 (26.9)	749 (33.0)	256 (34.8)	1,115 (31.9)	222 (26.4)	653 (33.3)	240 (34.8)
Age adjusted HR (95% Cl)		0.81 (0.69–0.94)	1.00 (ref)	1.06 (0.92–1.22)		0.79 (0.68–0.92)	1.00 (ref)	1.06 (0.91–1.23)
Fully adjusted HR (95% Cl)^b^		0.81 (0.70–0.96)	1.00 (ref)	1.06 (0.90–1.23)		0.80 (0.68–0.93)	1.00 (ref)	1.06 (0.90–1.24)
Cancer mortality								
Number (rate per 1,000 PY)	397 (9.16)	70 (7.9)	234 (8.97)	93 (11.1)	358 (8.9)	75 (7.9)	203 (9.0)	80 (10.2)
Age adjusted HR (95% Cl)		0.83 (0.64–1.09)	1.00 (ref)	1.26 (0.99–1.56)		0.85 (0.64–1.10)	1.00 (ref)	1.17 (0.90–1.50)
Fully adjusted HR (95% Cl)^b^		0.84 (0.63–1.11)	1.00 (ref)	1.26 (0.97–1.64)		0.86 (0.65–1.13)	1.00 (ref)	1.14 (0.87–1.50)
Fractures^d^								
Number (rate per 1,000 PY)	308 (14.3)	61 (13.8)	196 (15.2)	51 (12.0)	287 (14.4)	67 (14.1)	173 (15.5)	47 (11.9)
Age adjusted HR (95% Cl)		0.89 (0.67–1.19)	1.00 (ref)	0.80 (0.59–1.09)		0.90 (0.68–1.19)	1.00 (ref)	0.78 (0.56–1.07)
Fully adjusted HR (95% Cl)^b^		0.76 (0.57–1.04)	1.00 (ref)	0.98 (0.70–1.36)		0.78 (0.58–1.06)	1.00 (ref)	0.91 (0.65–1.28)
Women^e^								
SUA, median (IQR), mmol/L	0.31 (0.26–0.36)	0.23 (0.21–0.24)	0.31 (0.28–0.34)	0.43 (0.40–0.46)	0.31 (0.26–0.35)	0.23 (0.21–0.24)	0.31 (0.28–0.34)	0.41 (0.39–0.44)
All‐cause mortality								
Number (rate per 1,000 PY)	756 (13.7)	154 (12.9)	428 (12.9)	174 (17.5)	658 (13.1)	140 (13.2)	385 (12.4)	133 (15.1)
Age adjusted HR (95% Cl)		1.07 (0.89–1.29)	1.00 (ref)	1.27 (1.06–1.51)		1.13 (0.93–1.37)	1.00 (ref)	1.20 (0.99–1.46)
Fully adjusted HR (95% Cl)^b^		1.07 (0.88–1.30)	1.00 (ref)	1.10 (0.90–1.34)		1.10 (0.90–1.36)	1.00 (ref)	1.12 (0.91–1.39)
Disability‐free survival^c^								
Number (rate per 1,000 PY)	789 (19.4)	162 (18.4)	453 (18.5)	174 (23.8)	684 (18.4)	142 (18.2)	421 (18.4)	121 (18.6)
Age adjusted HR (95% Cl)		1.05 (0.88–1.26)	1.00 (ref)	1.19 (1.00–1.42)		1.03 (0.85–1.25)	1.00 (ref)	0.98 (0.80–1.20)
Fully adjusted HR (95% Cl)^b^		1.14 (0.94–1.37)	1.00 (ref)	0.99 (0.82–1.21)		1.10 (0.90–1.35)	1.00 (ref)	0.85 (0.69–1.05)
Cardiovascular disease								
Number (rate per 1,000 PY)	509 (9.92)	100 (8.96)	304 (9.84)	105 (11.3)	455 (9.65)	89 (8.97)	277 (9.56)	89 (10.8)
Age adjusted HR (95% Cl)		0.96 (0.76–1.20)	1.00 (ref)	1.06 (0.85–1.32)		0.99 (0.78–1.25)	1.00 (ref)	1.10 (0.87–1.39)
Fully adjusted HR (95% Cl)^b^		1.04 (0.82–1.33)	1.00 (ref)	0.92 (0.72–1.19)		1.04 (0.81–1.34)	1.00 (ref)	1.03 (0.80–1.33)
MACE								
Number (rate per 1,000 PY)	363 (7.03)	68 (6.05)	223 (7.19)	72 (7.71)	324 (6.83)	62 (6.21)	204 (7.01)	58 (7.00)
Age adjusted HR (95% Cl)		0.87 (0.67–1.15)	1.00 (ref)	1.00 (0.67–1.15)		0.92 (0.69–1.22)	1.00 (ref)	0.97 (0.73–1.30)
Fully adjusted HR (95% Cl)^b^		0.96 (0.72–1.29)	1.00 (ref)	0.83 (0.61–1.11)		0.97 (0.72–1.32)	1.00 (ref)	0.88 (0.64–1.20)
Dementia^c^								
Number (rate per 1,000 PY)	385 (7.44)	97 (8.67)	222 (7.11)	66 (7.04)	345 (7.26)	86 (8.65)	210 (7.19)	49 (5.85)
Age adjusted HR (95% Cl)		1.29 (1.02–1.64)	1.00 (ref)	0.94 (0.71–1.23)		1.26 (0.98–1.62)	1.00 (ref)	0.80 (0.59–1.09)
Fully adjusted HR (95% Cl)^b^		1.13 (0.87–1.46)	1.00 (ref)	1.05 (0.77–1.43)		1.13 (0.87–1.48)	1.00 (ref)	0.91 (0.66–1.27)
Cancer incidence								
Number (rate per 1,000 PY)	943 (19.0)	186 (17.2)	571 (19.1)	186 (20.9)	851 (18.7)	165 (17.2)	527 (18.8)	159 (20.0)
Age adjusted HR (95% Cl)		0.91 (0.77–1.07)	1.00 (ref)	1.08 (0.92–1.28)		0.92 (0.77–1.10)	1.00 (ref)	1.06 (0.89–1.27)
Fully adjusted HR (95% Cl)^b^		0.96 (0.80–1.14)	1.00 (ref)	0.98 (0.82–1.18)		0.95 (0.79–1.14)	1.00 (ref)	1.01 (0.84–1.23)
Cancer mortality								
Number (rate per 1,000 PY)	331 (6.02)	55 (4.61)	203 (6.13)	73 (7.34)	286 (5.67)	49 (4.63)	180 (5.81)	57 (6.46)
Age adjusted HR (95% Cl)		0.78 (0.58–1.05)	1.00 (ref)	1.15 (0.88–1.51)		0.82 (0.60–1.48)	1.00 (ref)	1.10 (0.81–1.48)
Fully adjusted HR (95% Cl)^b^		0.81 (0.59–1.11)	1.00 (ref)	0.95 (0.70–1.29)		0.79 (0.57–1.11)	1.00 (ref)	1.03 (0.74–1.42)
Fractures^d^								
Number (rate per 1,000 PY)	835 (31.3)	214 (37.8)	500 (31.2)	121 (24.4)	753 (30.9)	188 (37.5)	460 (30.7)	105 (24.2)
Age adjusted HR (95% Cl)		1.25 (1.07–1.47)	1.00 (ref)	0.75 (0.61–0.91)		1.26 (1.06–1.49)	1.00 (ref)	0.78 (0.63–0.97)
Fully adjusted HR (95% Cl)^b^		1.23 (1.04–1.46)	1.00 (ref)	0.77 (0.62–0.96)		1.22 (1.02–1.46)	1.00 (ref)	0.80 (0.64–1.00)

* IQR, interquartile range; HR, hazard ratio; Cl, confidence interval; MACE, major adverse cardiovascular events; PY, person‐years; Q, quintile; ref, reference; SUA, serum uric acid.

^a^ Study sample: total n = 5,126, Q1 n = 1,039, Q2–Q4 n = 3,082, Q5 n = 1,005; reference sample: total n = 4,723, Q n = 1,122, Q2–Q4 n = 2,666, Q5 n = 935.

^b^ Fully adjusted model: adjusted for age, body mass index, smoking status (current or former), current alcohol use, physical activity level, hypertension, diabetes, HDL‐C, non–HDL‐C, triglycerides, estimated glomerular filtration rate, and gamma‐glutamyl transpeptidase (>50 U/L).

^c^ Data from up to nine years of follow‐up.

^d^ Data for clinical trial only.

^e^ Study sample: total n = 6,320, Q1 n = 1,370, Q2–Q4 n = 3,793, Q5 n = 1,157; reference sample: total n = 5,778, Q1 n = 1,216, Q2–Q4 n = 3,546, Q5 n = 1,016.

## DISCUSSION

In this study, we established SUA reference intervals based on data from a relatively healthy population of individuals aged ≥70 years old. After adjusting for potential confounders, SUA levels in both the study and reference populations were not associated with a range of clinical events including dementia. In women only, however, low SUA levels were associated with a higher risk of fractures.

Existing reference intervals for SUA have been established primarily using data from middle‐aged populations across various ethnic groups (Supplementary Table 1). For instance, the Brazilian Longitudinal Study of Adult Health recently proposed SUA reference intervals in healthy adults aged 35 to 74 years old to range from 0.24 to 0.55 mmol/L for men and 0.17 to 0.41 mmol/L for women.^4^ Similarly, SUA reference intervals were established in a healthy Ethiopian population aged 18 to 60 years old that ranged from 0.16 to 0.41 mmol/L for men and 0.13 to 0.35 mmol/L for women.^5^ Only one study reported reference intervals in an exclusively older healthy geriatric Chinese population (aged 60–96 years), which were lower than those found in the present study: 0.18 to 0.46 mmol/L for men and 0.13 to 0.44 mmol/L for women.^6^ Variations in factors such as ethnicity, medication use, diet, and the presence of comorbidities may explain these differences. The revised SUA reference intervals presented in the current study, derived from a predominantly White population, are higher for both men and women than previously published intervals.

The baseline cross‐sectional analysis revealed associations between SUA levels and several baseline characteristics that may have confounded an unadjusted analysis. Participants in the highest quintile of SUA (Q5) exhibited a higher BMI, waist‐to‐height ratio, total cholesterol–to–HDL‐C ratio, and a greater prevalence of CKD, hypertension, antihypertensive medication use, and elevated liver enzymes (ALT, AST, and GGT). This data support previous research linking elevated SUA levels to an adverse cardiometabolic profile.^14^

In both the study and reference samples, after adjusting for cardiovascular risk factors, SUA levels were not significantly associated with clinical outcomes, including all‐cause mortality, DFS, CVD, MACE, cancer incidence or mortality, or dementia. In women, however, low SUA levels were associated with an increased risk of fractures. Although early studies on the relationship between SUA and fracture risk have produced inconsistent findings, more recent research supports a protective role of higher SUA levels. For example, Romero et al reported that in a cohort of women who were menopausal, higher SUA levels were associated with increased bone mineral density and a lower incidence of vertebral fractures, which is consistent with our findings.^15^ Although SUA is known to influence several biochemical pathways, including acting as an antioxidant at physiologic concentrations, the mechanisms linking SUA to bone strength and fracture risk remain to be elucidated.

Our findings align with recent research using an innovative approach (truncated minimum chi‐square) that continuously assesses reference intervals based on age, avoiding abrupt shifts between age groups.^16^ Their study confirmed that SUA levels increase with age and, consistent with our results, reported that average levels in women are lower than in men, although they converge at older ages. Additionally, they observed diurnal variation in SUA levels and highlighted differences in measurements across various analytical systems. These factors underscore the need for context‐specific interpretation of SUA levels in clinical practice.

The strengths of this study include the extensive characterization of the cohort at baseline, prolonged follow‐up, and adjudication of longitudinal outcomes by expert panels. The principal limitation is the lack of data on recurrent gout or urate stones, which are key clinical targets for treatment. In clinical practice, the SUA level targeted in treating gout is within the reference interval. The main contribution of this report is the reassurance it provides, that SUA levels within and near the proposed reference intervals are not significantly associated with increased morbidity or mortality. An important exception is the potential association between low SUA levels and an increased risk of fractures in women. This finding may have clinical relevance when treating older women who already have an elevated risk of fracture because of osteoporosis or osteopenia. Although the findings presented here are specific to individuals aged 70 years and older, limiting generalizability to younger populations, they provide valuable, age‐specific reference data for older adults. Additionally, the predominantly Caucasian population of European ancestry may limit the applicability of these findings to other ethnic groups.

In summary, the proposed reference interval for SUA in a predominantly White population aged ≥70 years old are 0.24 to 0.54 mmol/L for men and 0.19 to 0.48 mmol/L for women. Despite previous reports linking SUA levels with an increased incidence of various chronic diseases, our analysis identified no such relationships after adjusting for cardiovascular risk factors, except for an increased fracture risk in women with low SUA.

## AUTHOR CONTRIBUTIONS

All authors contributed to at least one of the following manuscript preparation roles: conceptualization AND/OR methodology, software, investigation, formal analysis, data curation, visualization, and validation AND drafting or reviewing/editing the final draft. As corresponding author, Dr Rickard confirms that all authors have provided the final approval of the version to be published and takes responsibility for the affirmations regarding article submission (eg, not under consideration by another journal), the integrity of the data presented, and the statements regarding compliance with institutional review board/Declaration of Helsinki requirements.

## Supporting information


**Disclosure form**.

**Data S1:** Supplementary Methods

**Supplementary Figure 1:** Distribution of serum uric acid levels by eGFR categories (<45 and ≥46 mL/min/1.73m^2^) in males and females.

**Supplementary Figure 2:** Distribution of serum uric acid values according to sex

**Supplementary Figure 3:** Distribution of serum uric acid according to age, diuretics, BMI and alcohol consumption for males in study sample (top) and reference sample (bottom)

**Supplementary Figure 4:** Distribution of serum uric acid according to age, diuretics, BMI and alcohol consumption for females in study sample (top) and reference sample (bottom)

**Supplementary Figure 5:** Association between baseline serum uric acid and the risk of fractures in males and females. Restricted cubic splines were used to plot risk of fractures according to baseline serum uric acid values. Blue dashed lines = 95% CI. Black line = adjusted hazard ratios. Green lines = serum uric acid reference intervals: males = 0.24‐0.54 mmol/L and females = 0.19‐0.48 mmol/L.

**Supplementary Table 1:** Recently published serum uric acid reference ranges

**Supplementary Table 2:** Distribution of serum uric acid values according to eGFR categories in all population, males and females.

**Supplementary Table 3:** Distribution of serum uric acid levels in males and females
