# Supplementary material for: Serum Uric Acid Levels in Older Adults: Associations With Clinical Outcomes and Implications for Reference Intervals in Those Aged 70 Years and Over
Source: Arthritis Care Res (Hoboken). 2025 Dec 17;78(3):407–16. doi: 10.1002/acr.25621 (PMC12975696; doi:10.1002/acr.25621)
Supplement: Supplementary file 2 — Data S1: Supplementary Methods [file ACR-78-407-s008.docx]

**Supplementary Methods**

**Recruitment:**

In **Australia**, ASPREE recruitment primarily occurred through general practices, with general practitioners (GPs) acting as co-investigators. Patient eligibility was assessed using clinical databases, and approved individuals received a study invitation from their GP, including a toll-free number for further inquiries. Interested participants underwent additional screening and, if suitable, attended a baseline visit at their GP's clinic. Following an interim visit to confirm eligibility, randomization occurred at a follow-up appointment four weeks later.

**Inclusion Criteria:**

Participants were eligible to take part in the study if they:

- Could provide informed consent
- Able attend a study visit
- Were male or female
- Were aged 70 years or older, with no upper age limit

**Exclusion Criteria:**

Participants were excluded from the study if they met any of the following conditions:

- History of cardiovascular or cerebrovascular disease defined as myocardial infarction, heart failure, angina pectoris, stroke, transient ischemic attack, carotid artery disease (≥50% stenosis) or prior carotid surgery/stenting, coronary procedures such as angioplasty/stenting or bypass surgery or abdominal aortic aneurysm
- Clinically diagnosed atrial fibrillation
- Cognitive impairment defined as a diagnosis of dementia or a score below 78 on the Modified Mini-Mental State Examination (3MS)
- Significant physical disability defined as severe difficulty or inability to independently perform any of the six Katz basic activities of daily living: bathing, transferring, toileting, dressing, eating, or walking across a room
- Increased bleeding risk, including medical conditions with a high current or recurrent bleeding risk or anemia (hemoglobin <12 g/dL for men, <11 g/dL for women)
- A life-limiting illness likely to cause death within five years, as judged by the participant’s GP or primary care physician
- Use of certain medications, including ongoing use of antiplatelet or anticoagulant drugs or aspirin use for secondary prevention
- Uncontrolled high blood pressure, defined as systolic ≥180 mmHg and/or diastolic ≥105 mmHg
- Unwillingness to stop routine aspirin use for primary prevention
- Poor medication adherence, indicated by taking less than 80% of pills during a 4-week placebo run-in period
- Current participation in an ongoing clinical trial
