# Supplementary material for: Serum Uric Acid Levels in Older Adults: Associations With Clinical Outcomes and Implications for Reference Intervals in Those Aged 70 Years and Over
Source: Arthritis Care Res (Hoboken). 2025 Dec 17;78(3):407–16. doi: 10.1002/acr.25621 (PMC12975696; doi:10.1002/acr.25621)
Supplement: Supplementary file 3 — Supplementary Figure 1: Distribution of serum uric acid levels by eGFR categories (<45 and ≥46 mL/min/1.73m2) in males and females. [file ACR-78-407-s003.docx]

**Supplementary Figure 1.** Distribution of serum uric acid levels by eGFR categories (<45 and ≥46 mL/min/1.73m^2^) in males and females.
