# Supplementary material for: Serum Uric Acid Levels in Older Adults: Associations With Clinical Outcomes and Implications for Reference Intervals in Those Aged 70 Years and Over
Source: Arthritis Care Res (Hoboken). 2025 Dec 17;78(3):407–16. doi: 10.1002/acr.25621 (PMC12975696; doi:10.1002/acr.25621)
Supplement: Supplementary file 4 — Supplementary Figure 2: Distribution of serum uric acid values according to sex [file ACR-78-407-s007.docx]

**Supplementary Figure 2.** Distribution of serum uric acid values according to gender
