# Supplementary material for: Serum Uric Acid Levels in Older Adults: Associations With Clinical Outcomes and Implications for Reference Intervals in Those Aged 70 Years and Over
Source: Arthritis Care Res (Hoboken). 2025 Dec 17;78(3):407–16. doi: 10.1002/acr.25621 (PMC12975696; doi:10.1002/acr.25621)
Supplement: Supplementary file 6 — Supplementary Figure 4: Distribution of serum uric acid according to age, diuretics, BMI and alcohol consumption for females in study sample (top) and reference sample (bottom) [file ACR-78-407-s010.docx]

**Supplementary Figure 4.** Distribution of serum uric acid according to age, diuretics, BMI and alcohol consumption for females in study sample (top) and reference sample (bottom)

| Study sample | | | |
| --- | --- | --- | --- |
| Age | **Diuretics** | **BMI** | **Alcohol: drinks per week** |
|  |  |  |  |
| Reference sample | | | |
|  |  |  |  |
