# Supplementary material for: Serum Uric Acid Levels in Older Adults: Associations With Clinical Outcomes and Implications for Reference Intervals in Those Aged 70 Years and Over
Source: Arthritis Care Res (Hoboken). 2025 Dec 17;78(3):407–16. doi: 10.1002/acr.25621 (PMC12975696; doi:10.1002/acr.25621)
Supplement: Supplementary file 7 — Supplementary Figure 5: Association between baseline serum uric acid and the risk of fractures in males and females. Restricted cubic splines were used to plot risk of fractures according to baseline serum uric acid values. Blue dashed lines = 95% CI. Black line = adjusted hazard ratios. Green lines = serum uric acid reference intervals: males = 0.24‐0.54 mmol/L and females = 0.19‐0.48 mmol/L. [file ACR-78-407-s005.docx]

**Supplementary Figure 5.** Association between baseline serum uric acid and the risk of fractures in males and females. Restricted cubic splines were used to plot risk of fractures according to baseline serum uric acid values. Blue dashed lines = 95% CI. Black line = adjusted hazard ratios. Green lines = serum uric acid reference intervals: males = 0.24-0.54 mmol/L and females = 0.19-0.48 mmol/L.

| **Males** | **Females** |
| --- | --- |
|  |  |
