# Supplementary material for: Serum Uric Acid Levels in Older Adults: Associations With Clinical Outcomes and Implications for Reference Intervals in Those Aged 70 Years and Over
Source: Arthritis Care Res (Hoboken). 2025 Dec 17;78(3):407–16. doi: 10.1002/acr.25621 (PMC12975696; doi:10.1002/acr.25621)
Supplement: Supplementary file 8 — Supplementary Table 1: Recently published serum uric acid reference ranges [file ACR-78-407-s004.docx]

**Supplementary Table 1.** Recently published serum uric acid reference ranges

| **Reference** | **Population** | **Age** | **Country** | **Reference interval** | **Exclusion criteria** |
| --- | --- | --- | --- | --- | --- |
| Bakrim, S., *et al.,* *Reference Intervals for Routine Biochemical Markers and Hematological Indices Derived from Healthy Adults in the Mediterranean Region of Morocco.* Clin Lab. 2023 Feb 1;69(2). | n= 768 | 18-60 years | Morocco | Males: 0.16 – 0.41 mmol/L  Females: 0.15 – 0.39 mmol/L | Cardiac or renal diseases, malnourished, diabetes mellitus, thyroid disease, anaemia, haemorrhagic haematological, or thrombotic disease histories, donated blood as a donor in the past 3 months, major surgery, drug ad- ministration, hypertension, cancer and chemotherapy, positive HBV (hepatitis B virus), positive HCV (hepatitis C virus), positive HIV (human immunodeficiency virus), positive syphilis (TPHA: Treponema Pallidum Hemagglutinations Assay and VDRL: Venereal Disease Research Laboratory), pregnant, smoker or chronic alcohol drinker. |
| Dório, M., *et al*., *Reference range of serum uric acid and prevalence of hyperuricemia: a cross-sectional study from baseline data of ELSA-Brasil cohort.* Adv Rheumatol. 2022 May 8;62(1):15. | n = 15,100 | 35-74 years | Brazil (Brazilian Longitudinal Study of Adult Health) | Men: 0.24 to 0.55 mmol/L  Women: 0.17 to 0.41 mmol/L  Considering only individuals with BMI < 25 kg/m2:  Men: 3.7 to 8.5 mg/100 ml  Women: 2.7 to 6.2 mg/100 ml | Individuals taking aspirin, thiazides, urate-lowering drugs and oestrogen replacement therapy, those reporting excessive alcohol intake or with estimated glomerular filtration rate < 60 ml/min/1.73 m2. |
| Abebe, M., *et al., Reference intervals of routine clinical chemistry parameters among apparently healthy young adults in Amhara National Regional State, Ethiopia.* PLoS One. 2018 Aug 2;13(8):e0201782. | n = 1175 | 18–60 years | Ethiopia | Males: 0.16 to 0.41 mmol/L  Females: 0.13 to 0.35 mmol/L | Individuals with diabetes mellitus, chronic renal insufficiency, hypertension, ischemic heart disease, anaemia, thyroid disease and liver diseases.  Individuals taking medications, chronic alcohol abusers, smokers, pregnant and lactating women, positive for transfusion transmissible infections (human immune deficiency virus (HIV), hepatitis B virus (HBV), hepatitis C virus (HCV) and syphilis), and who had a history of jaundice within 3 months and major surgery within 1 year. |
| Yang, Y., *et al*., *Reference intervals for serum bilirubin, urea, and uric acid in healthy Chinese geriatric population.* J Clin Lab Anal. 2018 Mar;32(3):e22318. | n = 820 | 60-96 years | China (healthy geriatric population) | Men: 0.18 to 0.46 mmol/L  Women: 0.13 to 0.44 mmol/L | A diagnosis of acute or chronic diseases; a history of surgery; a blood transfusion or blood donation within 4 months; excessive drinking (more than 30 g per day) and smoking (more than 20 cigarettes per day); blood pressure (systolic pressure ≥140 mm Hg and/or diastolic pressure ≥90 mm Hg; body mass index ≥28 kg/m2 or ≤18.5 kg/m2; triglyceride≥2.26 mmol/L, total cholesterol ≥6.22 mmol/L, fasting blood glucose >7.0mmol/L, hepatitis B surface antigen, anti- hepatitis C virus or anti-HIV positive. |
| Das, M., *et al*., *Reference Ranges for Serum Uric Acid among Healthy Assamese People.* Biochem Res Int. 2014;2014:171053. | n = 1470 | 35-86 years | India (Healthy adult Assamese population) | Men: 0.21 to 0.52 mmol/L  Women: 0.15 to 0.41 mmol/L | High blood sugar; high blood urea; high serum creatinine; derange LFT; high serum GGT; Lipaemia (high serum triglyceride, >200 mg/dL); High serum CRP (>0.6 mg/dL); High serum RA (≥20 IU/mL); Low blood haemoglobin (M: <13 gm/dL) (F: <11 gm/dL) |
| Sairam, S., *et al.*, *Hematological and biochemical parameters in apparently healthy Indian population: defining reference intervals.* Indian J Clin Biochem. 2014 Jul;29(3):290-7. | n = 10,665 | 20-70 years | India | Males: 0.21 to 0.49 mmol/L | Participants with known pathologic states, diabetes mellitus, chronic renal insufficiency, hypertension, ischemic heart disease, anaemia, thyroid disorders, liver diseases with biochemical and haematological abnormalities, weight loss, fever, chest pain, giddiness, polyarthralgia and loss of appetite, intake of pharmacologically active sub- stances, usage of drugs, multiple and regular vitamins, oral contraceptive pills, smokers, alcohol consumption more than two drinks per week (60 mL of alcohol), past illness of typhoid, tuberculosis, malaria, dengue within 6 months of the study date and jaundice or major surgery, pregnancy, lactating women and blood pressure (BP) C140/90 mm of Hg. |
| Saathoff, E., *et al*., *Laboratory reference values for healthy adults from southern Tanzania.* Trop Med Int Health. 2008 May;13(5):612-25. | n = 301 | 19 to 48 years | Tanzania | Males: 196 – 459 μmol/L  Females: 148 - 360 μmol/L  Males: 0.20 – 0.46 mmol/L  Females: 0.15 – 0.36 mmol/L | HIV-positive; pregnant or on medication; body temperature 37.5 C; physical examination or interview revealed other signs or symptoms of disease (e.g. malaria or other acute infections). |
