# Supplementary material for: Serum Uric Acid Levels in Older Adults: Associations With Clinical Outcomes and Implications for Reference Intervals in Those Aged 70 Years and Over
Source: Arthritis Care Res (Hoboken). 2025 Dec 17;78(3):407–16. doi: 10.1002/acr.25621 (PMC12975696; doi:10.1002/acr.25621)
Supplement: Supplementary file 9 — Supplementary Table 2: Distribution of serum uric acid values according to eGFR categories in all population, males and females. [file ACR-78-407-s006.docx]

**Supplementary Table 2.** Distribution of serum uric acid values according to eGFR categories in all population, males and females.

|  | **Serum uric acid values** | | |
| --- | --- | --- | --- |
| **eGFR categories (**mL/min/1.73m2) | **All** | **Males** | **Females** |
| <45 | 0.43 (0.38-0.50) | 0.46 (0.41-0.52) | 0.41 (0.36-0.49) |
| ≥46 | 0.34 (0.28-0.39) | 0.37 (0.33-0.43) | 0.31 (0.26-0.36) |
| P-value | <0.001 | <0.001 | <0.001 |
