# Supplementary material for: Serum Uric Acid Levels in Older Adults: Associations With Clinical Outcomes and Implications for Reference Intervals in Those Aged 70 Years and Over
Source: Arthritis Care Res (Hoboken). 2025 Dec 17;78(3):407–16. doi: 10.1002/acr.25621 (PMC12975696; doi:10.1002/acr.25621)
Supplement: Supplementary file 10 — Supplementary Table 3: Distribution of serum uric acid levels in males and females [file ACR-78-407-s001.docx]

**Supplementary Table 3.** Distribution of serum uric acid levels in males and females

|  | **Males** | **Females** |
| --- | --- | --- |
| Sample size | 5492 | 6386 |
| Mean, mmol/L (SD) | 0.38 (0.08) | 0.32 (0.08) |
| Range (low – high), mmol/L | 0.21-0.58 | 0.16-0.55 |
